# The profile and persistence of clinically critical antibiotic resistance genes and human pathogenic bacteria in manure-amended farmland soils

**DOI:** 10.3389/fcimb.2022.1073118

**Published:** 2022-11-24

**Authors:** Lin Zhu, Yulu Lian, Da Lin, Guoping Lin, Meizhen Wang

**Affiliations:** ^1^ School of Environmental Science and Engineering, Zhejiang Gongshang University, Hangzhou, China; ^2^ Zhejiang Provincial Key Laboratory of Solid Waste Treatment and Recycling, Hangzhou, China

**Keywords:** microbial contamination, manure-amended soils, clinically critical ARGs, human bacterial pathogens, virulence factor genes, “last-resort” antibiotics

## Abstract

**Introduction:**

Microbial contamination in farmlands is usually underestimated and understudied. Different fertilization times and manure origins might introduce and change the microorganism diversity in farmland soils and thus might influence the abundance and persistence of microbial contamination including antibiotic resistance genes (ARGs), human bacterial pathogens (HBPs), and virulence factor genes (VFGs).

**Methods:**

A 0.5-/1.5-year fertilization experiment was performed, and metagenomic sequencing was conducted to quantify microbial contamination. The resistomes of soil samples revealed that ARGs against antibiotics which were extensively used in veterinary medicine as well as clinically critical ARGs (CCARGs) persisted in manure-amended soils. Here the extended-spectrum beta-lactamase and carbapenemase bla genes, the high-level mobilized colistin resistance gene mcr, the tigecycline resistance gene tet(X), and the vancomycin resistance gene van, all of which can circumvent the defense line of these “last-resort” antibiotics were selected to investigate CCARG pollution in farm environments.

**Results:**

A total of 254 potential HBPs and 2106 VFGs were detected in soil samples. Overall, our results revealed that (1) farmland soils could serve as a reservoir of some important bla, mcr, tet(X), and van gene variants, (2) the diversity and relative abundance of HBPs and VFGs increased significantly with incremental fertilization times and were discrepant among different manureamended soils, and (3) most CCARGs and VFGs coexisted in HBPs.

**Disscusion:**

The results of this study suggested a biological risk of manure in spreading antimicrobial resistance and pathogenicity.

## Introduction

In the “One Health” model, although clinical use is the primary driver of the increase of antimicrobial resistance (AMR) in humans, the environment represents a significant AMR reservoir and an important route of antibiotic resistance gene (ARG) transmission to humans (Walsh, 2013). The United Nations Environment Programme (UNEP) has identified environmental AMR as one of six emerging concerns (UNEP, 2017). The World Health Organization (WHO) and the US Centers for Disease Control and Prevention (CDC) classify AMR pathogens as an imminent threat to human health (WHO, 2014; CDC, 2019). Therefore, as the AMR crisis escalates, extensive investigations must be conducted about the emergence of resistance and their relationship with pathogenicity in environmental reservoirs.

There is increasing interest in the role of manures in the spread of ARGs to soils, which are the source of these genes in the plant, aquatic, and wildlife ecosystems (Singer et al., 2016; Bengtsson-Palme et al., 2018). Therefore, animal manure must be considered as an important source of antibiotic resistance bacteria and the transmission of ARGs to the environment. The use of animal manure as fertilizer in farmlands and aquaculture is an important route of introduction of AMR into the soil and aquaculture. Although various studies have also analyzed the resistome of manure application, manures are not only highly contaminated with vet-relevant and common ARGs but also composed of a high number of clinically relevant ARGs. Therefore, manures pose a high potential ecological and health risk when applied to farmlands.

The resistome of soil samples revealed that ARGs against antibiotics which are widely used in veterinary medicine as well as clinically critical ARGs (CCARGs) persisted in manure-amended soils. Beta-lactam antibiotics (third-generation cephalosporins and carbapenems), polymyxin, and tigecycline are essential for the treatment of human Gram-negative bacterial infections (Zhu et al., 2021), and vancomycin is considered to be a “last-resort” therapy for severe infections that are caused by Gram-positive bacteria (Mühlberg et al., 2020). The excessive use of “last-resort” antibiotics will result in the emergence of “superbugs”, and ARGs against “last-resort” antibiotics are particularly crucial. The *bla*, *mcr*, *tet*(X), and *van* gene variants have diffused worldwide due to poor infectious control and a highly mobile and connected world population (Papp-Wallace et al., 2011). Here these clinically important ARGs were selected to investigate CCARG pollution in farmlands.

All hygienic and ecological risks related to the utilization of animal manures are complex. It has been reported that human bacterial pathogens (HBPs) are able to persist in animal manure under typical farming conditions (Venglovsky et al., 2009). A correlation between the prevalence of CCARGs and the HBPs in manure-amended farmland soils has not been reported.

Important knowledge remains a large gap regarding the contribution of manure to the emergence, accumulation, and risk of AMR and HBPs in different fertilization types and history. Furthermore, metagenomics-based methods have not been widely used to assess CCARG contamination in soil environments. This work comparatively quantifies the diversity and abundance of CCARGs and HBPs in different manure-amended farmland soils that are clinically important to assess the risk to human health. The persistence of CCARGs in the natural environment could complicate the diffusion dynamics of clinically critical drug-resistant HBPs, thus affecting the epidemiology and accelerating the rate of evolution of important antibiotic resistance bacteria. Thus, the co-occurrence network could provide us with new insights into the CCARGs, virulence factor genes (VFGs), and their potential HBP hosts.

## Materials and methods

### Sampling design and DNA extraction

Soil samples were collected from a long-term field that was an experiment managed by the Zhejiang Academy of Agricultural Sciences (120.42° N, 30.44° E) and located in Haining City, Zhejiang Province, China. Six different fertilization treatments were conducted on April, 2020: (1) background soil (BS), (2) 75 kg/mu fertilizer application soil (CK), (3) 500 kg/mu pig manure with 45 kg/mu of chemical fertilizer application soil (PM), (4) 500 kg/mu chicken manure with 45 kg/mu of chemical fertilizer application soil (CM), (5) 500 kg/mu cow dung with 45 kg/mu of chemical fertilizer application soil (CD), and 500 kg/mu silkworm excrement with 45 kg/mu of chemical fertilizer application soil (SE). The same amounts of fertilizers and manures were applied before cultivation in April 2021. Each topsoil was collected from the four corners and the centers after 0.5 years (0.5-a, 2020.10) and 1.5 years (1.5-a, 2021.10), generating a total of 33 soil samples. The details regarding fertilization time, sampling sites, and types of manure fertilizer are summarized in **Table 1**.

**Table 1:** Sampling setting information.

Sample name	Treatment	Fertilization time		Sampling time (year)	
BS	Background soil			2020-10 (0.5a)	2020-10 (1.5a)
CK	1,125 kg/ha of chemical fertilizer application soil	Apr-2020	Apr-2021	2020-10 (0.5)	2021-10 (1.5)
PM	7,500 kg/ha pig manure + 675 kg/ha of chemical fertilizer application soil	Apr-2020	Apr-2021	2020-10 (0.5)	2021-10 (1.5)
CM	7,500 kg/ha chicken manure + 675 kg/ha of chemical fertilizer application soil	Apr-2020	Apr-2021	2020-10 (0.5)	2021-10 (1.5)
CD	7,500 kg/ha cow dung + 675 kg/ha of chemical fertilizer application soil	Apr-2020	Apr-2021	2020-10 (0.5)	2021-10 (1.5)
SE	7,500 kg/ha silkworm excrement + 675 kg/ha of chemical fertilizer application soil	Apr-2020	Apr-2021	2020-10 (0.5)	2021-10 (1.5)

The FastDNA™ Spin Kit for soil (MP Biomedicals, USA) was used to extract the total genomic DNA from 0.5 g of soil and fertilizer samples. The concentration and the purity of DNA were measured using a Nano2000 (Thermo Fisher Scientific, MA, USA), USA) and Qubit fluorometer (Invitrogen, USA), respectively. The high-quality DNA was stored in an ultra-low-temperature freezer for subsequent analysis (Bastian et al., 2009).

### Illumina shotgun metagenomic sequencing

For each sample, approximately 5 mg of qualified DNA was used for library construction (350 bp) and then sequenced on the Illumina Hiseq 2000 platform (Illumina Inc., USA, PE150 strategy) by the Majorbio Bio-pharm Technology Co., Ltd. (Shanghai, China). Approximately 12 Gb of raw reads were generated for each sample. All of the raw reads were filtered using fastp software (https://github.com/OpenGene/fastp) to trim or remove the low-quality reads with the default parameters (Chen et al., 2018). After conducting quality control procedures, approximately 6–12 Gb of metagenomic sequences were generated for each soil sample to yield a total of 210 Gb of data. The related information of metagenomic datasets for each sample is listed in **Supplementary Table S1**. All of the data were analyzed using the online platform Majorbio I-Sanger Cloud Platform (www.i-sanger.com).

### Detection of ARGs based on reads

The ARG sequences were annotated by online-analysis pipeline ARGs-OAP v2.0 (SARGfam database: http://smile.hku.hk/SARGs) with a sequence identity of 90% and alignment length of more than 25 amino acids at cutoff E-value ≤ 1 ×10^-7^ (Yin et al., 2018). The SARG database contained 24 ARG types (representing the class of antibiotics to which ARGs confer resistance) and 1,244 ARG subtypes (representing individual kinds of ARGs). The abundances of ARGs types/subtypes were calculated as “copies of ARG per prokaryote’s cell” (copies per cell) after normalization of the cell numbers.

### Bacteria community and HBP analysis

To characterize the microbial community structure among all samples, the clean reads from each sample were assembled into contigs by Megahit (Li et al., 2015c), and predicted open reading frames were aligned against the NCBI non-redundant (http://www.ncbi.nlm.nih.gov) protein database using DIAMOND with E-value < 1 × 10^-5^ (Buchfink et al., 2015). The abundance of bacterial species was analyzed at each taxonomic level for the construction of the abundance table at the corresponding taxonomic level.

The HBPs and dangerous HBPs were screened using the bacterial pathogen database list as described previously (Zhu et al., 2022), which was combined with a list of bacterial pathogens from a previous study (Li et al., 2015a), the Virulence Factor Database (VFDB; http://www.mgc.ac.cn/VFs/) (Chen et al., 2005; Liu et al., 2022) and Pathogen Host Interactions (PHI-base) (Urban et al., 2020). The list is presented in **Supplementary Table S2**.

### Characterization of VFGs

Broad spectrum detection of VFGs was conducted using VFDB (latest updated in 2022) using blastx (E-value cutoff ≤ 1×10^-10^). If hit sequences were searched against CARD with a coverage ≥ 90% and identity ≥ 90%, they were considered to be VFG-like sequences, where all of the data in the VFDB were divided into 14 categories (level 1) including over 100 subcategories and 2,132 VFGs (Liu et al., 2022).

### Statistical analysis and network analysis

The averages and standard deviations were calculated using Microsoft Excel 2016 (Microsoft Office, Microsoft, USA). The histogram, scatter diagram, and heat map were generated by OriginLab 2022 (OriginLab Corporation, USA). Principal coordinate analysis (PCoA) was conducted by R software (version 3.6.1) with the Vegan package and ggplot2 to evaluate the difference between ARG and HBP abundance in the soil samples. The correlation was statistically calculated using IBM SPSS statistical soft version 24, and the results were subjected to *t*-test statistical analysis for significance, which were indicated with asterisks: *P < 0.05 and **P < 0.01. Networks were rendered using Gephi 0.9.1 based on a significant Pearson correlation (*r* > 0.7, *P* < 0.05) for CCARGs, dangerous HBPs, and VFGs.

### Availability of data and materials

The sequence data have been deposited to NCBI Sequence Read Archive database BioProject (accession no. PRJNA407411).

## Results

### Broad-spectrum profile of ARG in soils with a different fertilization history and manure origin

**Figure 1:**
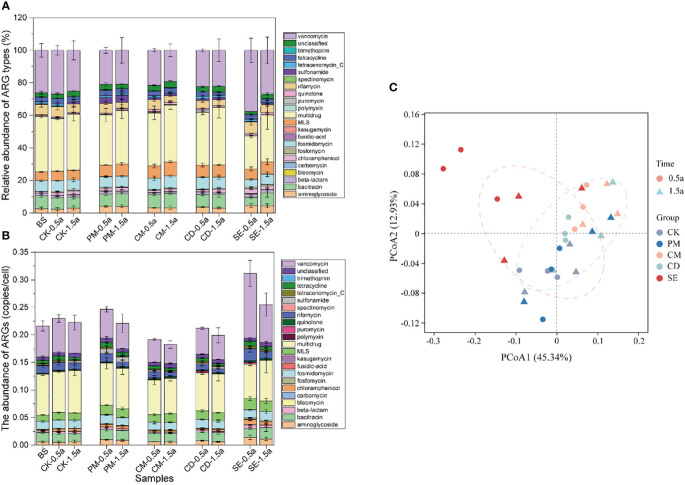
Profile of antibiotic resistance genes (ARG) types from different manure-amended soils at 0.5 and 1.5 years. **(A)** Percentage of ARG types in different samples. **(B)** Relative abundance of ARG types in different samples. **(C)** Principal coordinate analysis showing the overall distribution pattern of ARG types among different samples. BS, background soil; CK, chemical fertilizer application soil; PM, pig manure application soil; CM, chicken manure application soil; CD, cow dung application soil; SE, silkworm excrement. .

The metagenomics analysis of the soil samples revealed that multidrug (6.87 × 10^-2^ gene copies/cell, 30.21%), vancomycin (5.63 × 10^-2^ gene copies/cell, 24.77%), and bacitracin (1.72 × 10^-2^ gene copies/cell, 7.57%) resistance genes were the dominant ARG types in manure-amended farmlands (**Figure 1A**). The number of identified ARG subtypes were 205, 227, 188, and 190 in the SE, PM, CM, and CD soils, respectively, at the 0.5-year sampling time. At the 1.5-year sampling time, the number of identified ARGs were 228, 224, 212, and 200 in the SE, PM, CM, and CD soils, respectively (**Supplementary Figure S1**). The number of detected ARGs increased with growing sampling time. In contrast, the relative abundance of ARGs decreased with growing sampling time. Specifically, the average ARG abundance ranged from 0.19 to 0.31 copies/cell at the 0.5-year sampling time and 0.18–0.25 copies/cell at the 1.5-year sampling time for the four manure-amended soil samples. The highest abundance was in the SE sample, and the lowest abundance was in the CM sample (**Figure 1B**). The abundances of ARGs in the SE samples were significantly higher (*P* < 0.05) than those in other samples. The PCoA analysis revealed that the composition of ARGs in SE samples differed from those in other samples (**Figure 1C**). A total of 358 different ARGs belonging to 23 types were identified in soil samples, of which 109 types (109/354, 30.4%) were shared in all manure-amended soils, with an average abundance of 1.24 ×10^-3^–1.63 ×10^-3^ copies/cell (**Supplementary Table S3**).

### Contamination of manure-amended soils with CCARGs

To evaluate the urgency of the problem regarding CCARGs in manure-amended farmlands, the extended-spectrum beta-lactamase (ESBL) and carbapenemase genes, the high-level mobilized colistin resistance gene *mcr*, the tigecycline resistance gene *tet*(X), and the vancomycin resistance gene *van* were screened for using the SARG database. These genes are related to human health issues and the environmental transmission of ARGs.

**Figure 2:**
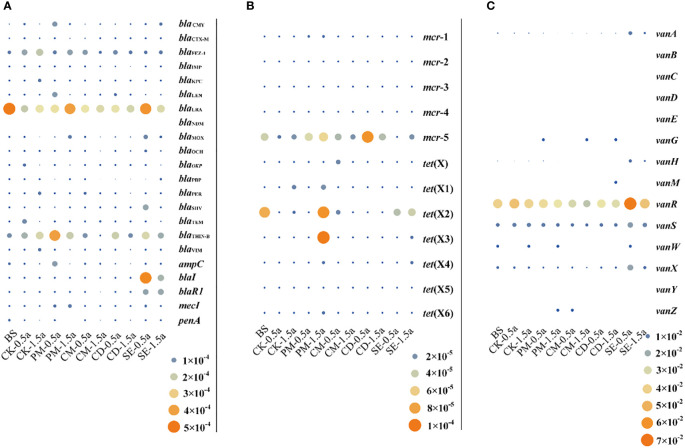
Bubble graph showing the relative abundance of clinically critical antibiotic resistance genes. **(A)** Relative abundance of extended-spectrum beta-lactamase and carbapenemase genes. **(B)** Relative abundance of mobilized colistin resistance genes and tigecycline resistance genes. **(C)** Relative abundance of vancomycin resistance genes.

ESBL and carbapenemases develop resistance by hydrolyzing beta-lactams, including penicillin, carbapenem, and cephalosporin. As shown in **Figure 2A**, 21 different beta-lactam resistance genes were distinctly present in manure-amended soils. Surprisingly, the most prevalent clinically relevant beta-lactamase genes, *bla*_NDM_ and *bla*_KPC_, were almost undetectable in soils. The most abundant ESBL and carbapenemase genes were *bla*_LRA_ and *bla*_THIN-B_ with average abundances of 2.87 × 10^−4^ and 1.67 × 10^−4^ copies/cell, respectively. The *bla*_CTX-M_ type beta-lactamase was previously the most prevalent type of ESBL (Bonnet, 2004), with a relatively low abundance of 5.18 × 10^−6^ copies/cell among detected ESBL and carbapenemase genes in manure-amended soils. The average relative abundance of other ESBL and carbapenemase genes ranged from 4.96 × 10^−6^ to 9.09 × 10^−5^ copies/cell. Moreover, SE and PM soil samples possessed the most abundant ESBL and carbapenemase genes, with average values of 5.08 × 10^−5^ and 4.71 × 10^−5^ copies/cell, respectively.

The development of polymyxin resistance is directly linked to its agricultural use as well as in human antibiotics with polymyxin being used in animal production in several countries (Hao et al., 2014). Mobilized colistin resistance gene *mcr* gene variants have become disseminated by lateral/horizontal diffusion into diverse environments, including aquatic, soil, plant, wildlife, animal, and medical environments. The *mcr*-1 gene was only detected in PM samples, with a relative abundance of 6.86 × 10**^−^** ^6^. The *mcr*-2–*mcr*-4 genes were not detected in any of the samples, while *mcr*-5 was detected in all samples. The average abundances of *mcr*-5 genes were 1.59 × 10**^−^** ^5^, 5.01 × 10**^−^** ^5^, 2.64 × 10**^−^** ^5^, 6.08 × 10**^−^** ^5^, and 1.02 × 10**^−^** ^5^ copies/cell in CK, PM, CM, CD, and SE samples, respectively (**Figure 2B**). Furthermore, an increasing number of *tet*(X) gene variants comprise an expanding family of tetracycline (tigecycline)-inactivating enzyme, and except for *tet*(X5), other *tet*(X) gene variants could be identified in soils. The *tet*(X) gene (1.71 × 10**^−^** ^5^ copies/cell) and the *tet*(X5) genes (9.09 × 10**^−^** ^5^ copies/cell) were only detected in CM and PM samples, respectively. The *tet*(X2) gene was the most abundant gene variant and was detected in BS, CK, PM, CM, and SE samples. Moreover, PM samples were important reservoirs with the most diversity and abundance of *tet*(X) gene variants and contained *tet*(X1)–*tet*(X4) and *tet*(X6), all of which were detected in SE samples with an average abundance of 2.35 × 10^−5^ copies/cell (**Figure 2B**).

The relative abundances of vancomycin resistance genes were much higher than those of the abovementioned three types of genes. The *van*A, *van*B, *van*C, *van*D. *van*E, *van*H, *van*R, *van*S, *van*X, and *van*Y genes had 100% detection rates. The *van*R and *van*S genes were dominant gene types in the group of vancomycin resistance genes, with average abundances of 3.88 × 10^−2^ and 8.97 × 10^−3^ copies/cell, respectively. The average abundances of the other ARGs against vancomycin were 1.90 × 10^−5^ and 4.86 × 10^−3^ copies/cell. The relative abundance of *tet*(X) genes in SE samples (6.62 × 10^−3^ copies/cell) was the highest among the six different treatments (**Figure 2C**).

### Diversity and abundance of HBPs in manure-amended soils

The results obtained demonstrated similar taxa distribution patterns with a highly uneven community that was predominated by several bacterial phyla among all samples. Proteobacteria was the most abundant phylum, accounting for more than 50% of the microbial composition among the samples. The differences in microbial community composition among the samples were primarily due to the proportion of Proteobacteria and Actinobacteria present (**Supplementary Figure S2**).

**Figure 3:**
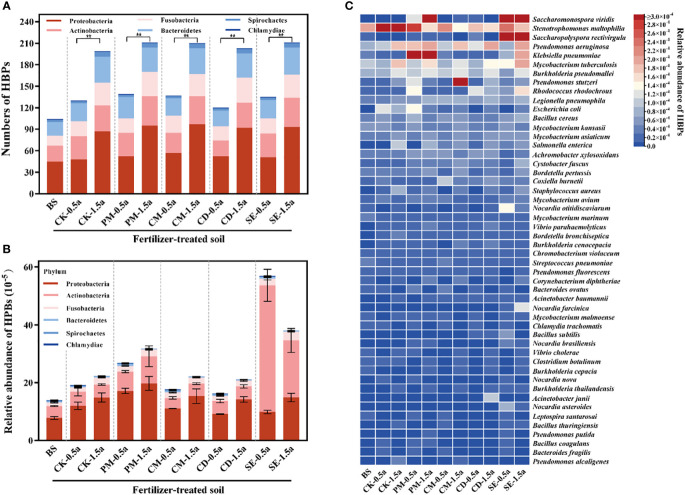
Diversity and abundance of human bacterial pathogens (HBPs) among different soils. **(A)** Number of detected HBPs. **(B)** Relative abundance of HBPs. **(C)** Heat map showing the abundance of HBPs that are detected in different samples. Significant differences between two treatment groups with **P < 0.01.

The number of potential HBPs detected increased significantly with incremental fertilization times (*P* < 0.05) (**Figure 3A**). The potential HBP phyla include Proteobacteria (1.33 × 10^−4^), Actinobacteria (9.99 × 10^−5^), Fusobacteria (2.05 × 10^−5^), Bacteroidetes (3.33 × 10^−5^), Spirochaetes (2.26 × 10^−6^), and Chlamydiae (1.17 × 10^−6^). Proteobacteria (51.1%) and Actinobacteria (38.4%) were the dominant potential HBPs in farmland soils (**Figure 3B**). A total of 254 species of potential HBPs were detected in all 33 soil samples using the previous bacterial pathogen database list (**Supplementary Table S4**) (Zhu et al., 2022). *Saccharomonospora viridis* was the most dominant potential HBP (**Figure 3C**). In addition, potential HBPs could be detected at levels of 5.69 × 10^−4^ (SE-0.5a)/3.81 × 10^−4^ (SE-1.5a) in SE samples and 2.68 × 10^−4^ (PM-0.5a)/3.17 × 10^−4^ (PM-1.5a) in PM samples at a higher abundance than in other samples.

**Figure 4:**
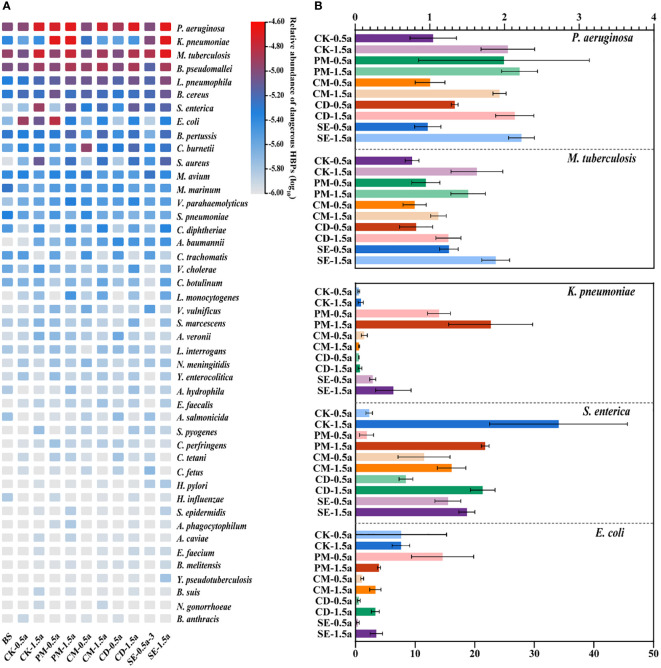
Persistence of dangerous human bacterial pathogens (HBPs) among all samples. **(A)** Abundance of 54 dangerous HBPs in different soil samples. **(B)** Fold changes of the dangerous HBPs [significantly different from background soil (BS)] compared with the BS.

As shown in **Figure 4A**, a total of 53 dangerous HBPs were identified in our samples using the VFDB, the WHO priority ARB list, and the emerging/re-emerging pathogens list (Zhu et al., 2022). *Pseudomonas aeruginosa* (1.46 × 10^−5^), *Klebsiella pneumoniae* (1.45 × 10^−5^), *Mycobacterium tuberculosis* (1.23 × 10^−5^), *Burkholderia pseudomallei* (1.01 × 10^−5^), and *Legionella pneumophila* (6.39 × 10^−6^) were the top five most dangerous HBPs, which represent a global threat to human health by their presence in the food chain or by direct exposure. The fold change value of some dangerous HBPs (*P. aeruginosa*, *M. tuberculosis*, *K. pneumoniae*, *Salmonella enterica*, and *Escherichia coli*) showed that the application of manures resulted in the presence of these dangerous HBPs in manure-amended soils compared with the unfertilized BS samples (**Figure 4B**). Compared with the BS, *S. enterica* exhibited the greatest difference among the different samples, with an increase that was greater than 10-fold in most samples.

### Diversity and abundance of VFGs in manure

**Figure 5:**
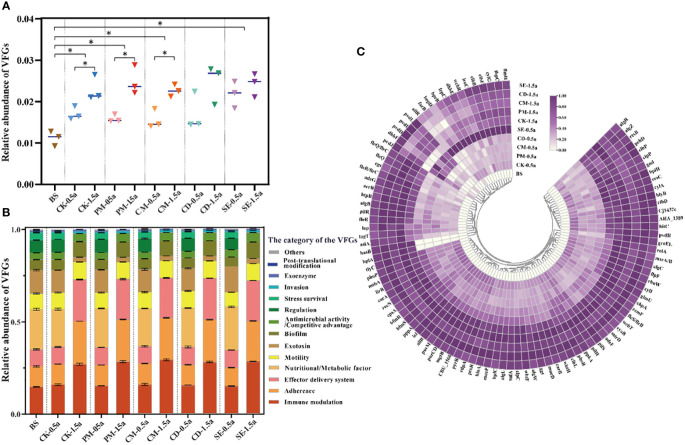
Virulence factor gene (VFG) profiles impacted by different manures in soils. **(A)** Total relative abundance of VFG types. **(B)** Proportion of VFG type abundance in different samples. **(C)** Log-normalized abundance of the top 100 VFGs in different samples. Significant differences between two treatment groups with *P < 0.05.

Similar to the distribution of HBPs, the VFG diversity and abundance increased in 1.5a-samples compared to 0.5a-samples, except for the CM samples. The abundance in the SE samples (0.0228 ± 0.0026) was the highest, followed by the abundance in the CD (0.0207 ± 0.0053) and PM (0.0204 ± 0.0052) samples (**Figure 5A**). Immune modulation VFGs were the dominant type (22.3% on average) in all samples (**Figure 5B**), followed by adherence VFGs (17.4% on average) and effector delivery system VFGs (16.4% on average). In total, 2,106 (2,106/2,232, 94.4%) genes were screened for various virulence factors. The top 100 abundant VFGs in the 1.5a-samples were also considerably higher in the 0.5a-samples (**Figure 5C**). Dominant types included nutritional/metabolic factors (25 subtypes), immune modulation (14 subtypes), and exotoxin (12 subtypes) types. Nutritional/metabolic factors with dominant subtypes include *fbp*C, *hit*C, mgtB, *car*B, and *pvd*H, which can be carried by *Neisseria meningitidis*, *Haemophilus influenzae*, *S. enterica*, *Francisella tularensis*, and *P. aeruginosa*. Immune modulators including *tuf*A, *wcb*R, *msb*A, *wbt*F, *wbt*H, *wcb*T, *glm*U, *cps*A, and *gnd* have as possible hosts *Francisella tularensis*, *Burkholderia pseudomallei*, *H. influenzae*, *Enterococcus faecalis*, and *K. pneumoniae*. Exotoxin VFGs including *cyl*G, *clb*F, *ces*C, *clb*B, and *rtx*B were carried by *Streptococcus agalactiae*, *K. pneumoniae*, *Bacillus cereus*, and *Vibrio cholerae* (**Figure 5C**, **Supplementary Table S5**).

### Co-occurrence patterns among CCARGs, HBPs, and VFGs

The relationship between resistomes and pathogenicity was assessed using the Pearson’s correlation of the total profiles of ARGs and HBPs. The total abundance of ARGs was positively related to HBPs (**Supplementary Figure S3**, Pearson’s *r* = 0.9202, *P* < 0.001) for 0.5-year samples and for 1.5-year samples (**Supplementary Figure S3**, Pearson’s *r* = 0.7122, *P* < 0.001).

**Figure 6:**
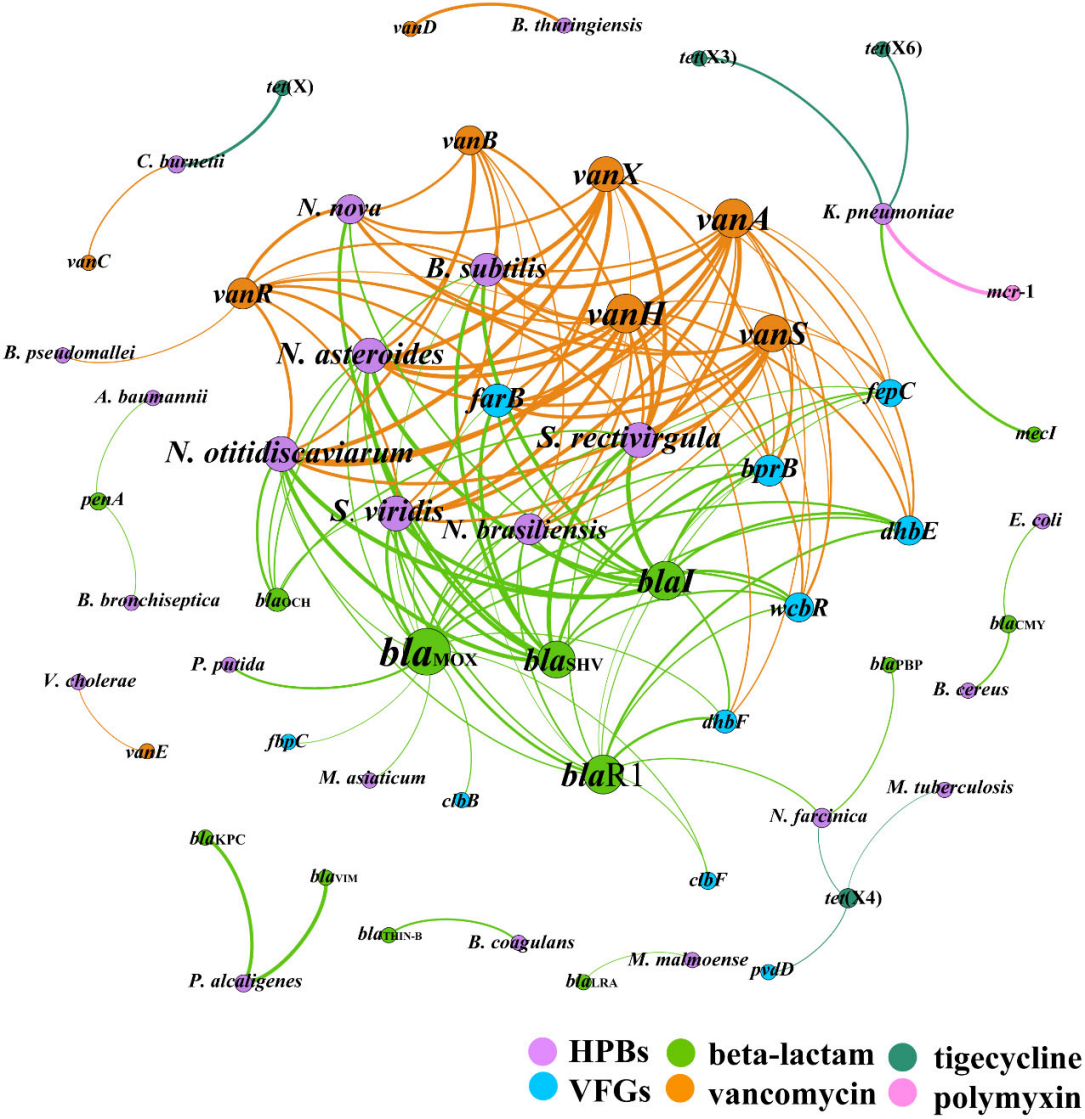
Network of co-occurring clinically critical antibiotic resistance genes, human bacterial pathogens, and virulence factor genes. A node represents each element, and a connection (*i*.*e*., edge) represents a significant (*P* < 0.05) and strong (Pearson’s *r* > 0.70) pairwise correlation.

To address the co-occurrence of CCARGs, HBPs, and VFGs, a network analysis was conducted. As shown in **Figure 6**, 147 pairs (*i*.*e*., edges) and strong (Pearson’s *r* > 0.70) and significant (*P* < 0.05) correlations were identified among 49 CCARGs and 50 dangerous HBPs and 50 VFG subtypes with CCARGs (*i*.*e*., 60 nodes; **Supplementary Table S3** shows the list of all elements used for the network analysis) in all samples. The network could indicate possible HBP host information for CCARGs. Strikingly, the beta-lactam and vancomycin resistance gene variants dominated the ARG types in the core network. They could coexist with many dangerous HBPs, such as *Nocardia asteroids*, *N. brasiliensis*, *N. otitidiscaviarum*, *S. rectivirgula*, and *B. subtilis*. These also harbored multiple VFGs including *far*B, *fep*C, *bpr*B, *bla*_KPC_, and *bla*_VIM_ that could coexist with *Pseudomonas alcaligenes*. High-risk HBP *K. pneumoniae* had a significantly strong correlation with the CCARGs *mec*I, *mcr*-1, *tet*(X3), and *tet*(X6).

## Discussion

In order to comprehensively investigate the profiles of microbial contamination in manure-amended soils, metagenomic shotgun high-throughput sequencing was executed to detect CCARGs, HBPs, and VFGs. It is widely recognized that the environment plays a big role in the transmission of CCARGs (Fletcher, 2015; Bengtsson-Palme et al., 2018; Tiedje et al., 2019). Previous studies demonstrated that the ESBL/MCR/Tet(X)-encoding genes, *i*.*e*., CCARGs, are ubiquitous in the farm environment (Lee et al., 2020; Fu et al., 2021; Shi et al., 2021). To the best of our knowledge, this study is the first to examine the changes in CCARGs and HBPs in different manure-amended soils with different times.

Beta-lactam antibiotics (third-generation cephalosporins and carbapenems) have been widely used as therapeutic and prophylactic drugs in animal-derived foods. The data indicate that animals used 3,236 tons of beta-lactam in 2018 alone (Sun et al., 2019). The beta-lactam genes are present in high diversity and abundance in soils. Colistin has been used in livestock for decades around the world. It was also used on a large scale before China banned its use as an animal growth promoter in 2017 (EMA, 2016), with 2,875 metric tons being used in animal production in 2014 (Shi et al., 2021). Thus, the *mcr* genes persistently existed in the farm environment (Shi et al., 2021). The *mcr*-1–*mcr*-10 genes have been identified in isolated strains from and/or directly in environmental samples (Anyanwu et al., 2020). Our previous study also reported the prevalence of *mcr* in aquatic environments, including watersheds and wastewaters (Zhu et al., 2020; Zhu et al., 2021), and the current study demonstrated that *mcr* is a core resistance gene in soil environments, indicating that contamination by colistin resistance genes is widespread in nature and anthropogenic environments. Here *mcr*-1 and *mcr*-5 were detected, and the absence of *mcr*-2, *mcr*-3, and *mcr*-4 in manure-amended soils was demonstrated. The *mcr*-1 gene was formerly the most prevalent *mcr* gene in more than 40 countries (Wang et al., 2017), and *mcr*-1 was present in relatively low amounts in our soil samples. The aquatic environment may be the major reservoir for *mcr*-3, and *Aeromonas* species potentially served as the most likely reservoir for this gene (Shen et al., 2018). In comparison with *mcr-*3 gene abundances, the *mcr-*5 gene was found much more frequently and in higher concentrations in soils. Epidemiological studies have shown that *mcr*-5 is one of the most prevalent members of the *mcr* family (Ling et al., 2020) and is widely distributed in various Gram-negative bacterial species.

The use of tigecycline in animals is not authorized; the high abundance of *tet*(X) may be attributed to the wide use of tetracycline. The *tet*(X2) variant may be the major *tet*(X) variant in these soil samples. Vancomycin is considered to the antibiotic of “last resort” for the treatment of related Gram-positive pathogen infections, some strains of which are resistant to most other antibiotics, and vancomycin has been prescribed cautiously over the past few decades (McKenna, 2013). Previous studies have shown that many ARGs which were resistant to vancomycin were detected in pig feces (Zhu et al., 2013). However, the abundance of vancomycin resistance genes was remarkably lower than that of widely used antibiotics in water environments (Li et al., 2015b). Unexpectedly, a high level of detection was observed for vancomycin resistance genes in soils. Overall, some CCARGs changed markedly across samples, suggesting that selective pressure on CCARGs varies from manure types and fertilization times. Pig manure and silkworm excrement application soils had a significantly higher abundance of CCARGs compared with chicken manure and cow dung application soils. Further treatment and monitoring of these persistent CCARGs would be necessary to prevent dissemination. The plasmid transfer of CCARGs means that they can be easily transported to many organisms in soils, including those that are clinically significant. Such transmissibility events may happen during manure or storage processing from HBPs to manure inherent microorganisms or vice versa.

Metagenomic sequencing is a powerful and promising approach for the comprehensive analysis of bacterial pathogenicity and virulence in the environment (Huang et al., 2018). In this study, the metagenomic data revealed that seven species (*P. aeruginosa*, *K. pneumoniae*, *M. tuberculosis*, *B. pseudomallei*, *L. pneumophila*, *S. enterica*, and *E. coli*) were the major dangerous pathogens in manure-amended soils, not only because of their high abundance but also because of their greater difference (high fold change) compared with BS. *P. aeruginosa* was one of the most infamous HBPs present in the group of highly virulent and drug-resistant bacterial pathogens. The lethal *K. pneumoniae* is usually related to serious infections caused by carbapenem-resistant *K. pneumoniae.* Valid antimicrobial options are usually scarce, and therapy often requires reliance on drugs with a risk of toxicity (*e*.*g*., polymyxin) or other safety concerns (*e*.*g*., tigecycline) (Kaewpoowat and Ostrosky-Zeichner, 2015). The abundance of *E. coli* is a definitive indicator of fecal contamination, and high counts pose a public health risk and promote the transmissibility of infectious diseases (Bhatt et al., 2021). Most of these HBPs belong to the group of antimicrobial-resistant ESKAPE pathogens (*Enterococcus faecium*, *Staphylococcus aureus*, *K. pneumoniae*, *Acinetobacter baumannii*, *P. aeruginosa*, and *Enterobacter species*) (Rice, 2008), which were designated “priority status” in the list of HBPs for which new AMR development is urgently needed (WHO, 2017). ESKAPE pathogens were important not only because they caused the greatest number of nosocomial infections but also because they represent a paradigm of pathogenicity, transmission, and resistance (Rice, 2008). Therefore, the presence of these high-risk HBPs in farmland soils deserves more attention.

A correlation-based network explored the co-occurrence patterns of CCARG-dangerous HBP–VFGs in all soil samples and helped to determine the risk assessment of CCARG acquisition by HBPs in manure-amended soils. The network revealed relatively strong correlation coefficients (*r >*0.7, *P* < 0.05) between multiple CCARGs, dangerous HBPs, and VFG subtypes in all samples. These co-occurrence patterns will pose a severe threat to ecological security and public health, particularly to manure handlers, crop farmers, and consumers of agricultural products from farms that are fertilized with these manures.

The prevalence of CCARGs has aroused extensive attention even beyond the biological and medical sciences, and their dissemination in the environment is also a great concern. This study suggests that long-term monitoring and surveillance of the persistence of CCARGs and HBPs in manure-amended soils are required. The study also provides quantitative data for specific recommendations for manure treatment, handling, and application procedures to translate them into practical on-farm management decisions to ultimately reduce the risk of human exposure in the future.

## Data availability statement

The datasets presented in this study can be found in online repositories. The names of the repository/repositories and accession number(s) can be found in the article/**Supplementary Material**.

## Author contributions

LZ and MW conceived and designed the experiments. LZ and DL collected samples. LZ and YL performed the experiments. LZ, YL, DL, GL, and MW analyzed the data. LZ wrote the manuscript. MW reviewed and edited the manuscript and offered the funding source. All authors contributed to the article and approved the submitted version.

## Funding

This work was supported by the National Key R&D Program of China (grant number 2018YFE0110500) and the National Natural Science Foundation of China (grant numbers 22122607, U21A20292, and 22076167).

## Conflict of interest

The authors declare that the research was conducted in the absence of any commercial or financial relationships that could be construed as a potential conflict of interest.

## Publisher’s note

All claims expressed in this article are solely those of the authors and do not necessarily represent those of their affiliated organizations, or those of the publisher, the editors and the reviewers. Any product that may be evaluated in this article, or claim that may be made by its manufacturer, is not guaranteed or endorsed by the publisher.

## References

[B1] Anyanwu M. U. Jaja I. F. Nwobi O. C. (2020). Occurrence and characteristics of mobile colistin resistance (mcr) gene-containing isolates from the environment: A review. Int. J. Environ. Res. Public Health 17, 1028. doi: 10.3390/ijerph17031028 32041167 PMC7036836

[B2] Bastian M. Heymann S. Jacomy M. (2009). Gephi: an open source software for exploring and manipulating networks. ICWSM 8, 361–362. doi: 10.1609/icwsm.v3i1.13937

[B3] Bengtsson-Palme J. Kristiansson E. Larsson D. G. J. (2018). Environmental factors influencing the development and spread of antibiotic resistance. FEMS Microbiol. Rev. 42, fux053. doi: 10.1093/femsre/fux053 29069382 PMC5812547

[B4] Bhatt D. L. Szarek M. Pitt B. Cannon C. P. Leiter L. A. McGuire D. K. . (2021). Sotagliflozin in patients with diabetes and chronic kidney disease. N. Engl. J. Med. 384, 129–139. doi: 10.1056/NEJMoa2030186 33200891

[B5] Bonnet R. (2004). Growing group of extended-spectrum beta-lactamases: the CTX-m enzymes. Antimicrob. Agents Chemother. 48, 1–14. doi: 10.1128/AAC.48.1.1-14.2004 14693512 PMC310187

[B6] Buchfink B. Xie C. Huson D. H. (2015). Fast and sensitive protein alignment using DIAMOND. Nat. Methods 12, 59–60. doi: 10.1038/nmeth.3176 25402007

[B7] C.D.C (2019) Antibiotic resistant threats in the united states 2019. Available at: https://www.cdc.gov/drugresistance/pdf/threats-report/2019-ar-threats-report-508.pdf

[B8] Chen L. Yang J. Yu J. Yao Z. Sun L. Shen Y. . (2005). VFDB: a reference database for bacterial virulence factors. Nucleic Acids Res. 33, D325–D328. doi: 10.1093/nar/gki008 15608208 PMC539962

[B9] Chen S. Zhou Y. Chen Y. Gu J. (2018). Fastp: an ultra-fast all-in-one FASTQ preprocessor. Bioinformatics 34, i884–i890. doi: 10.1093/bioinformatics/bty560 30423086 PMC6129281

[B10] EMA (2016). Updated advice on the use of colistin products in animals within the European union: Development of resistance and possible impact on human and animal health (EMA/CVMP/CHMP/231573/2016) (Amsterdam: EMA).

[B11] Fletcher S. (2015). Understanding the contribution of environmental factors in the spread of antimicrobial resistance. Environ. Health Prev. Med. 20, 243–252. doi: 10.1007/s12199-015-0468-0 25921603 PMC4491066

[B12] Fu Y. Chen Y. Liu D. Yang D. Liu Z. Wang Y. . (2021). Abundance of tigecycline resistance genes and association with antibiotic residues in Chinese livestock farms. J. Hazard. Mater. 409, 124921. doi: 10.1016/j.jhazmat.2020.124921 33421874

[B13] Hao H. Cheng G. Iqbal Z. Ai X. Hussain H. I. Huang L. . (2014). Benefits and risks of antimicrobial use in food-producing animals. Front. Microbiol. 5, 288. doi: 10.3389/fmicb.2014.00288 24971079 PMC4054498

[B14] Huang K. Mao Y. Zhao F. Zhang X. X. Ju F. Ye L. . (2018). Free-living bacteria and potential bacterial pathogens in sewage treatment plants. Appl. Microbiol. Biotechnol. 102, 2455–2464. doi: 10.1007/s00253-018-8796-9 29396586

[B15] Kaewpoowat Q. Ostrosky-Zeichner L. (2015). Tigecycline : a critical safety review. Expert Opin. Drug Saf. 14, 335–342. doi: 10.1517/14740338.2015.997206 25539800

[B16] Lee S. Mir R. A. Park S. H. Kim D. Kim H. Y. Boughton R. K. . (2020). Prevalence of extended-spectrum beta-lactamases in the local farm environment and livestock: challenges to mitigate antimicrobial resistance. Crit. Rev. Microbiol. 46, 1–14. doi: 10.1080/1040841X.2020.1715339 31976793

[B17] Li B. Ju F. Cai L. Zhang T. (2015a). Profile and fate of bacterial pathogens in sewage treatment plants revealed by high-throughput metagenomic approach. Environ. Sci. Technol. 49, 10492–10502. doi: 10.1021/acs.est.5b02345 26252189

[B18] Li D. Liu C. M. Luo R. Sadakane K. Lam T. W. (2015c). MEGAHIT: an ultra-fast single-node solution for large and complex metagenomics assembly *via* succinct de bruijn graph. Bioinformatics 31, 1674–1676. doi: 10.1093/bioinformatics/btv033 25609793

[B19] Ling Z. R. Yin W. J. Shen Z. Q. Wang Y. Shen J. Z. Walsh T. R. (2020). Epidemiology of mobile colistin resistance genes mcr-1 to mcr-9. J. Antimicrob. Chemo. 75, 3087–3095. doi: 10.1093/jac/dkaa205 32514524

[B20] Liu B. Zheng D. Zhou S. Chen L. Yang J. (2022). VFDB 2022: a general classification scheme for bacterial virulence factors. Nucleic Acids Res. 50, D912–D917. doi: 10.1093/nar/gkab1107 34850947 PMC8728188

[B21] Li B. Yang Y. Ma L. Ju F. Guo F. Tiedje J. M. . (2015b). Metagenomic and network analysis reveal wide distribution and co-occurrence of environmental antibiotic resistance genes. ISME J. 9, 2490–2502. doi: 10.1038/ismej.2015.59 25918831 PMC4611512

[B22] McKenna M. (2013). Antibiotic resistance: the last resort. Nature 499, 394–396. doi: 10.1038/499394a 23887414

[B23] Mühlberg E. Umstätter F. Kleist C. Domhan C. Mier W. Uhl P. (2020). Renaissance of vancomycin: approaches for breaking antibiotic resistance in multidrug-resistant bacteria. Can. J. Microbiol. 66, 11–16. doi: 10.1139/cjm-2019-0309 31545906

[B24] Papp-Wallace K. M. Endimiani A. Taracila M. A. Bonomo R. A. (2011). Carbapenems: past, present, and future. Antimicrob. Agents Chemother. 55, 4943–4960. doi: 10.1128/AAC.00296-11 21859938 PMC3195018

[B25] Rice L. B. (2008). Federal funding for the study of antimicrobial resistance in nosocomial pathogens: no ESKAPE. J. Infect. Dis. 197, 1079–1081. doi: 10.1086/533452 18419525

[B26] Shen Y. Xu C. Sun Q. Schwarz S. Ou Y. Yang L. . (2018). Prevalence and genetic analysis of mcr-3-Positive aeromonas species from humans, retail meat, and environmental water samples. Antimicrob. Agents Chemother. 62, e00404–00418. doi: 10.1128/AAC.00404-18 29967026 PMC6125509

[B27] Shi X. Li Y. Yang Y. Shen Z. Cai C. Wang Y. . (2021). High prevalence and persistence of carbapenem and colistin resistance in livestock farm environments in China. J. Hazard. Mater. 406, 124298. doi: 10.1016/j.jhazmat.2020.124298 33168321

[B28] Singer A. C. Shaw H. Rhodes V. Hart A. (2016). Review of antimicrobial resistance in the environment and its relevance to environmental regulators. Front. Microbiol. 7, 1728. doi: 10.3389/fmicb.2016.01728 27847505 PMC5088501

[B29] Sun H. Jiang Z. Shen X. Xu S. (2019). “Report on the use of veterinary antibiotics in China in 2018,” in The 15th Symposium of Veterinary Pharmacology and toxicology branch of Chinese animal husbandry and Veterinary Association. Ministry of Agriculture and Rural Affairs of China, Beijing.

[B30] Tiedje J. M. Wang F. Manaia C. M. Virta M. Sheng H. Liping M. A. . (2019). Antibiotic resistance genes in the human-impacted Environment:A one health perspective. Pedosphere 029, 273–282. doi: 10.1016/S1002-0160(18)60062-1

[B31] U.N.E.P (2017) Antimicrobial resistance from environmental pollution among biggest emerging health threats, says UN Environment|UN environment. Available at: https://www.unenvironment.org/news-and-stories/press-release/antimicrobial-resistance-environmental-pollution-among-biggest (Accessed 4 August 2019).

[B32] Urban M. Cuzick A. Seager J. Wood V. Rutherford K. Venkatesh S. Y. . (2020). PHI-base: the pathogen-host interactions database. Nucleic Acids Res. 48, D613–D620. 31733065 10.1093/nar/gkz904 PMC7145647

[B33] Venglovsky J. Sasakova N. Placha I. (2009). Pathogens and antibiotic residues in animal manures and hygienic and ecological risks related to subsequent land application. Bioresour. Technol. 100, 5386–5391. doi: 10.1016/j.biortech.2009.03.068 19386485

[B34] Walsh F. (2013). The multiple roles of antibiotics and antibiotic resistance in nature. Front. Microbiol. 4, 255. doi: 10.3389/fmicb.2013.00255 23986757 PMC3753432

[B35] Wang Y. Tian G. B. Zhang R. Shen Y. Tyrrell J. M. Huang X. . (2017). Prevalence, risk factors, outcomes, and molecular epidemiology of mcr-1-positive enterobacteriaceae in patients and healthy adults from China: an epidemiological and clinical study. Lancet Infect. Dis. 17, 390–399. doi: 10.1016/S1473-3099(16)30527-8 28139431

[B36] W.H.O (2014) Antimicrobial resistance: global report on surveillance 2014. Available at: https://www.who.int/antimicrobial-resistance/publications/surveillancereport/en/

[B37] W.H.O (2017). Global priority list of antibiotic-resistant bacteria to guide research, discovery, and development of new antibiotics. Available at: http://www.who.int/medicines/publications/WHO-PPL-Short_Summary_25Feb-ET_NM_WHO.pdf?ua=1

[B38] Yin X. Jiang X. T. Chai B. Li L. Yang Y. Cole J. R. . (2018). ARGs-OAP v2.0 with an expanded SARG database and hidden Markov models for enhancement characterization and quantification of antibiotic resistance genes in environmental metagenomes. Bioinformatics 34, 2263–2270. doi: 10.1093/bioinformatics/bty053 29408954

[B39] Zhu Y. G. Johnson T. A. Su J. Q. Qiao M. Guo G. X. Stedtfeld R. D. . (2013). Diverse and abundant antibiotic resistance genes in Chinese swine farms. Proc. Natl. Acad. Sci. U.S.A. 110, 3435–3440. doi: 10.1073/pnas.1222743110 23401528 PMC3587239

[B40] Zhu L. Lian Y. Lin D. Huang D. Yao Y. Ju F. . (2022). Insights into microbial contamination in multi-type manure-amended soils: The profile of human bacterial pathogens, virulence factor genes and antibiotic resistance genes. J. Hazard. Mater. 437, 129356. doi: 10.1016/j.jhazmat.2022.129356 35728317

[B41] Zhu L. Shuai X. Y. Lin Z. J. Sun Y. J. Zhou Z. C. Meng L. X. . (2021). Landscape of genes in hospital wastewater breaking through the defense line of last-resort antibiotics. Water Res. 209, 117907. doi: 10.1016/j.watres.2021.117907 34864622

[B42] Zhu L. Zhou Z. Liu Y. Lin Z. Shuai X. Xu L. . (2020). Comprehensive understanding of the plasmid-mediated colistin resistance gene mcr-1 in aquatic environments. Environ. Sci. Technol. 54, 1603–1613. doi: 10.1021/acs.est.9b05919 31886662

