# Supplementary material for: The profile and persistence of clinically critical antibiotic resistance genes and human pathogenic bacteria in manure-amended farmland soils
Source: Front Cell Infect Microbiol. 2022 Nov 24;12:1073118. doi: 10.3389/fcimb.2022.1073118 (PMC9729351; doi:10.3389/fcimb.2022.1073118)
Supplement: Supplementary file 1 [file DataSheet_1.docx]

**The profile and persistence of clinically critical antibiotic resistance genes and human pathogenic bacteria in manure-amended farmland soils**

Lin Zhu^a^, Yulu Lian^a^, Da Lin^a^, Guoping Lin ^a^, Meizhen Wang^a,b,*^

*^a^ School of Environmental Science and Engineering, Zhejiang Gongshang University, Hangzhou 310012, China*

*^b^ Zhejiang Provincial Key Laboratory of Solid Waste Treatment and Recycling, Hangzhou 310012, China*

**Corresponding author:**

Meizhen Wang

PhD, Professor

School of Environmental Science and Engineering, Zhejiang Gongshang University

Tel: +86-571-2887-7174

E-mail: wmz@mail.zjgsu.edu.cn


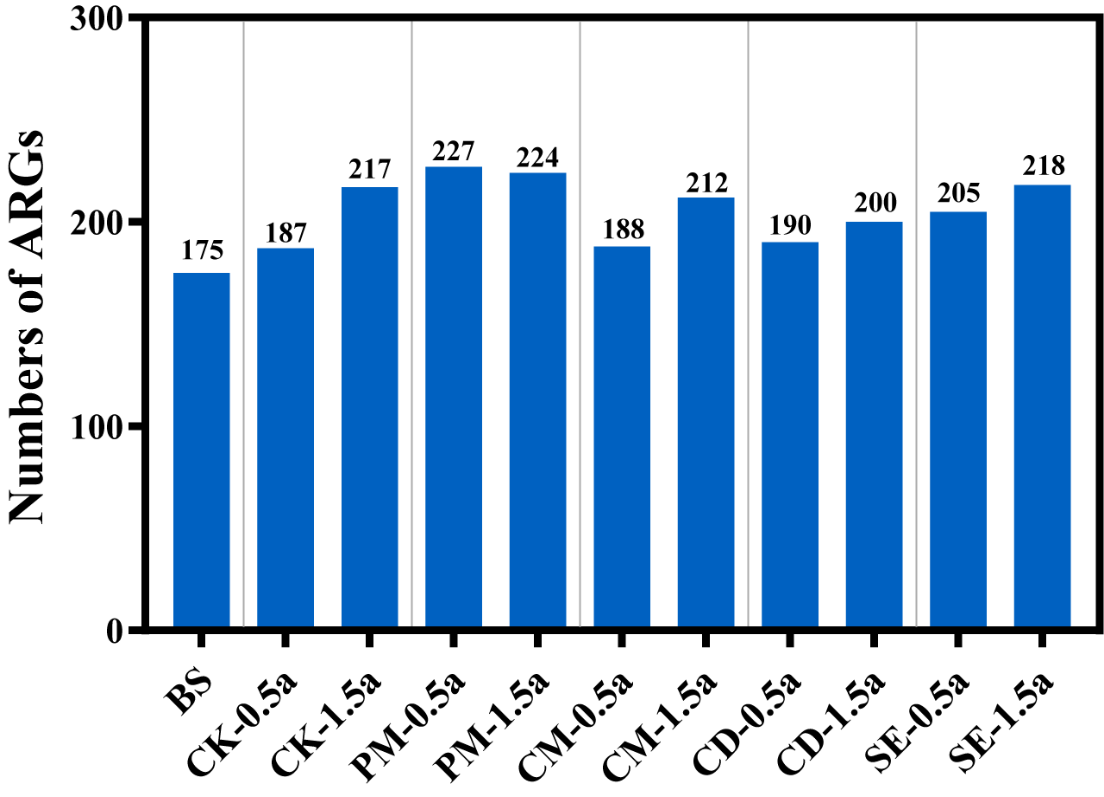


**Fig. S1 The number of detected ARGs in all samples**


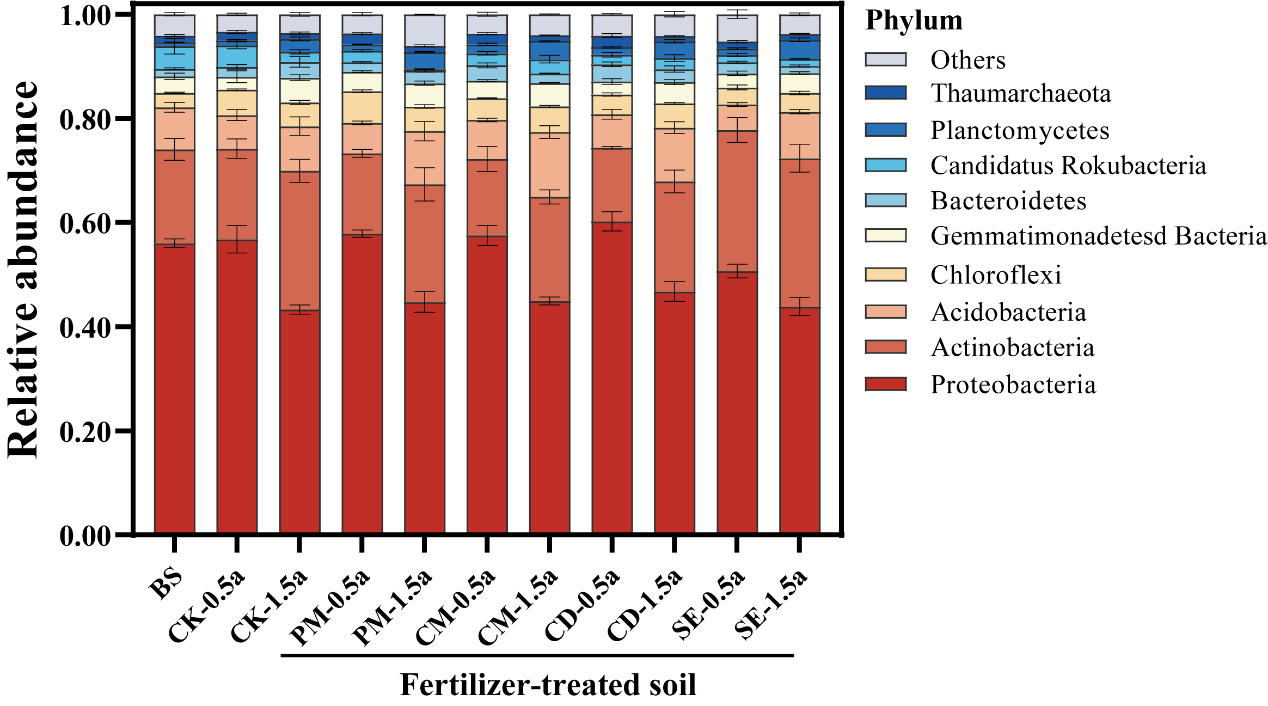


**Fig. S2 Microbial community composition at the phylum level for all samples**


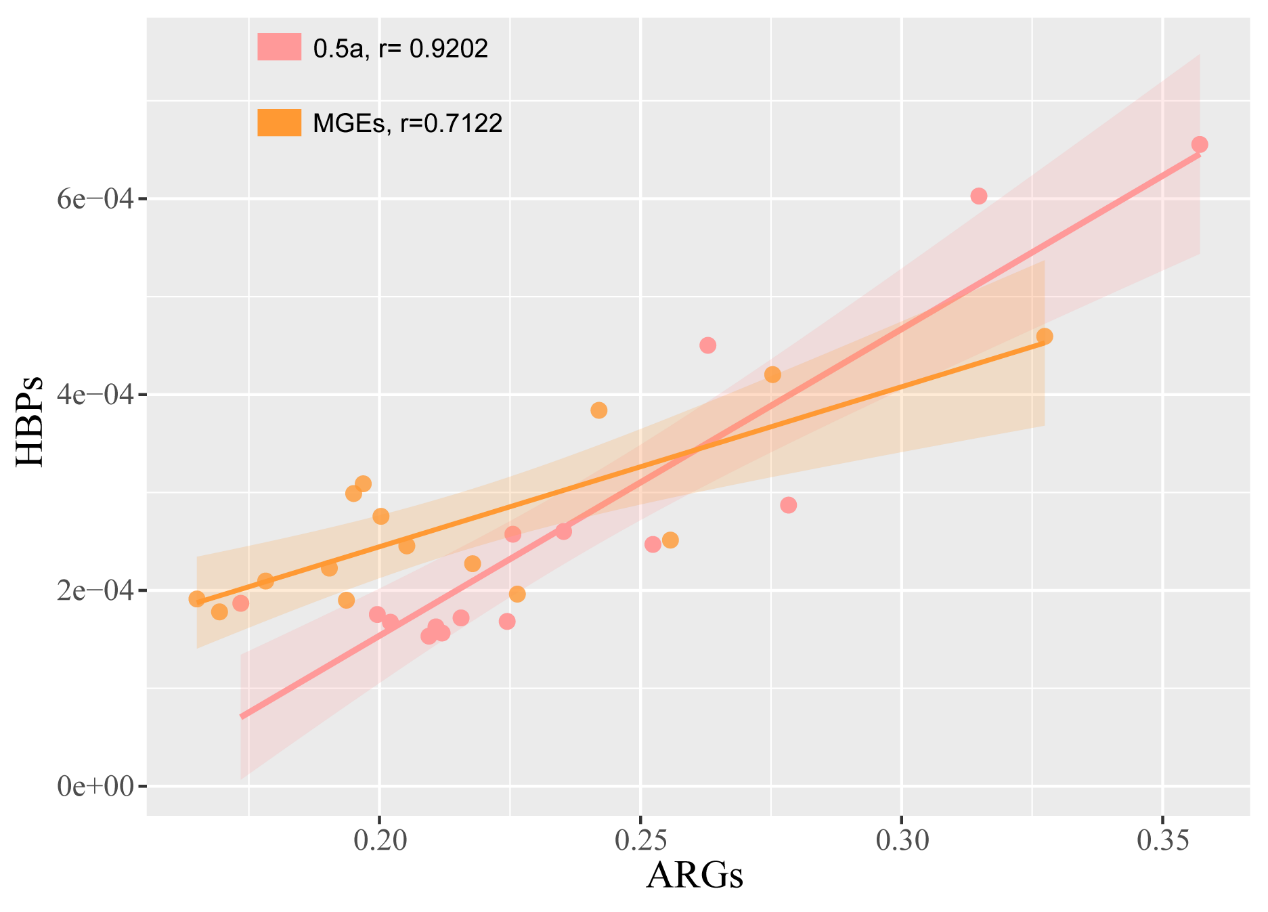


**Fig. S3 The correlation of ARGs and HBPs at different sampling time**
